# Predictors of complete obliteration and favourable outcome after single-modality treatment for low-grade arteriovenous malformations: a multicentre study

**DOI:** 10.1093/esj/aakag103

**Published:** 2026-08-31

**Authors:** Angelica M Fuentes, Salem M Tos, Natasha Ironside, Georgios Mantziaris, Yuki Shinya, Basel Musmar, Hamza Adel Salim, Nimer Adeeb, Christopher S Ogilvy, Douglas Kondziolka, Ali Alaraj, Min S Park, Adam A Dmytriw, Hussein Zeineddine, Finn Mccarthy, Hussam Abou-Al-Shaar, Ahmed Abdelsalam, Mustafa Baskaya, Cagdas Ataoglu, Anthony Sanchez-Forteza, Muhammed Amir Essibayi, Abdullah Keles, Sandeep Muram, Howard Riina, Arwin Rezai, Sahin Hanalioglu, Ufuk Erginoglu, Johannes Pöppe, Davide Simonato, Yan-Lin Li, Sandeep Kandregula, Dhairya A Lakhani, Christoph J Griessenauer, Rahim Abo Kasem, Alejandro M Spiotta, Ajit S Puri, Jasmeet Singh, Anna Luisa Kuhn, Jan Karl Burkhardt, Robert M Starke, Laligam N Sekhar, Michael R Levitt, David Altschul, Neil Haranhalli, Malia McAvoy, Paul Foreman, Osama O Zaidat, Mohammad H AlMajali, Hakeem J Shakir, Alfred Pokmeng See, Adib A Abla, Aashay Patel, Andrew Nguyen, Matthew J Koch, Visish M Srinivasan, Peng Roc Chen, Spiros Blackburn, Ketan R Bulsara, Peter Kan, Louis Kim, Omar Choudhri, Stavropoula I Tjoumakaris, Pascal Jabbour, Amey Savardekar, Hugo H Cuellar, Michael T Lawton, Bharat Guthikonda, Jacques Morcos, Jason P Sheehan

**Affiliations:** Department of Neurosurgery, University of Virginia, Charlottesville, VA, United States; Department of Neurosurgery, University of Virginia, Charlottesville, VA, United States; Department of Neurosurgery, University of Virginia, Charlottesville, VA, United States; Department of Neurosurgery, University of Virginia, Charlottesville, VA, United States; Department of Neurosurgery, University of Virginia, Charlottesville, VA, United States; Department of Neurosurgery, Thomas Jefferson University Hospital, Philadelphia, PA, United States; Department of Neuroradiology, MD Anderson Medical Center, Houston, TX 77030, United States; Department of Radiology, Louisiana State University, New Orleans, LA, United States; Department of Neurosurgery, Louisiana State University, New Orleans, LA, United States; Neurological Service, BIDMC, Harvard Medical School, Boston, MA, United States; Department of Neurosurgery, New York University Grossman School of Medicine, New York, NY, United States; Department of Neurosurgery, University of Illinois in Chicago, Chicago, IL, United States; Department of Neurosurgery, Loyola University Stritch School of Medicine, Maywood, IL, United States; Neuroendovascular Program, Massachusetts General Hospital & Brigham and Women’s Hospital, Harvard Medical School, Boston, MA, United States; Neurointerventional & Neuroanalytics Collaboration (NAN-C), School of Medicine, Toronto Metropolitan University, Toronto, Ontario, Canada; Department of Neurosurgery, UT Health Sciences Center at Houston, McGovern Medical School, Houston, TX, United States; Department of Neurosurgery, University of Illinois in Chicago, Chicago, IL, United States; Department of Neurosurgery, UT Health Sciences Center at Houston, McGovern Medical School, Houston, TX, United States; Department of Neurosurgery, University of Miami, Miami, FL, United States; Department of Neurosurgery, University of Wisconsin School of Medicine, Madison, WI, United States; Department of Neurosurgery, University of Wisconsin School of Medicine, Madison, WI, United States; Department of Neurosurgery, University of Illinois in Chicago, Chicago, IL, United States; Montefiore Einstein Cerebrovascular Research Lab and Department of Neurological Surgery, Montefiore Medical Center, Albert Einstein College of Medicine, New York, NY, United States; Department of Neurosurgery, University of Wisconsin School of Medicine, Madison, WI, United States; Neurological Service, BIDMC, Harvard Medical School, Boston, MA, United States; Department of Neurosurgery, New York University Grossman School of Medicine, New York, NY, United States; Department of Neurosurgery, Christian Doppler Klinik, Paracelsus Medical University, Salzburg, Austria; Department of Neurosurgery, Brigham and Women Hospital, Harvard Medical School, Boston, MA, United States; Department of Neurosurgery, University of Wisconsin School of Medicine, Madison, WI, United States; Department of Neurosurgery, Christian Doppler Klinik, Paracelsus Medical University, Salzburg, Austria; Department of Neuroradiology, Oxford University Hospitals NHS Foundation Trust, Nuffield Department of Surgical Sciences, University of Oxford, Oxford, United Kingdom; Department of Neuroradiology, Oxford University Hospitals NHS Foundation Trust, Nuffield Department of Surgical Sciences, University of Oxford, Oxford, United Kingdom; Department of Neurosurgery, University of Pennsylvania, Philadelphia, PA, United States; Department of Neuroradiology, Rockefeller Neuroscience Institute, West Virginia University, Morgantown, WV, United States; Department of Neurosurgery, Christian Doppler Klinik, Paracelsus Medical University, Salzburg, Austria; Department of Neurosurgery, Division of Neuroendovascular Surgery, Medical University of South Carolina, Charleston, SC, United States; Department of Neurosurgery, Division of Neuroendovascular Surgery, Medical University of South Carolina, Charleston, SC, United States; Division of Neurointerventional Radiology, Department of Radiology, University of Massachusetts Medical Center, Worcester, MA, United States; Division of Neurointerventional Radiology, Department of Radiology, University of Massachusetts Medical Center, Worcester, MA, United States; Division of Neurointerventional Radiology, Department of Radiology, University of Massachusetts Medical Center, Worcester, MA, United States; Department of Neurosurgery, University of Pennsylvania, Philadelphia, PA, United States; Department of Neurosurgery, University of Miami, Miami, FL, United States; Department of Neurosurgery, University of Washington, Seattle, WA, United States; Department of Neurosurgery, University of Washington, Seattle, WA, United States; Montefiore Einstein Cerebrovascular Research Lab and Department of Neurological Surgery, Montefiore Medical Center, Albert Einstein College of Medicine, New York, NY, United States; Montefiore Einstein Cerebrovascular Research Lab and Department of Neurological Surgery, Montefiore Medical Center, Albert Einstein College of Medicine, New York, NY, United States; Department of Neurosurgery, University of Washington, Seattle, WA, United States; Department of Neurosurgery, Orlando Health Neuroscience, Orlando, FL, United States; Department of Neurosurgery, Mercy Health, St Vincent Medical Center, Bridgeport, CT, United States; Department of Neurosurgery, Mercy Health, St Vincent Medical Center, Bridgeport, CT, United States; Department of Neurosurgery, University of Oklahoma Health Sciences Center, Oklahoma City, OK, United States; Department of Neurosurgery, Boston Children’s Hospital, Boston, MA, United States; Department of Neurosurgery, University of Miami, Miami, FL, United States; Department of Neurosurgery, University of Florida, Gainesville, FL, United States; Department of Neurosurgery, University of Florida, Gainesville, FL, United States; Department of Neurosurgery, University of Florida, Gainesville, FL, United States; Department of Neurosurgery, University of Pennsylvania, Philadelphia, PA, United States; Department of Neurosurgery, UT Health Sciences Center at Houston, McGovern Medical School, Houston, TX, United States; Department of Neurosurgery, UT Health Sciences Center at Houston, McGovern Medical School, Houston, TX, United States; Department of Neurosurgery, University of Connecticut, Mansfield, CT, United States; Department of Neurosurgery, University of Texas Medical Branch, Galveston, TX, United States; Department of Neurosurgery, University of Washington, Seattle, WA, United States; Department of Neurosurgery, University of Pennsylvania, Philadelphia, PA, United States; Department of Neurosurgery, Thomas Jefferson University Hospital, Philadelphia, PA, United States; Department of Neurosurgery, Thomas Jefferson University Hospital, Philadelphia, PA, United States; Department of Neurosurgery, Louisiana State University, New Orleans, LA, United States; Department of Neurosurgery, Louisiana State University, New Orleans, LA, United States; Department of Neurosurgery, Barrow Neurological Institute, Phoenix, AZ, United States; Department of Neurosurgery, Louisiana State University, New Orleans, LA, United States; Department of Neurosurgery, UT Health Sciences Center at Houston, McGovern Medical School, Houston, TX, United States; Department of Neurosurgery, University of Virginia, Charlottesville, VA, United States

**Keywords:** AVM obliteration, cerebral arteriovenous malformations, complication rates, endovascular, functional outcomes, resection, Spetzler–Martin grade I and II, stereotactic radiosurgery

## Abstract

**Introduction:**

Patient and arteriovenous malformation (AVM) characteristics that portend success after treatment for low-grade brain AVMs remain unknown.

**Patients and methods:**

We utilised the MISTA multicentre registry to identify patients with Spetzler–Martin (SM) grade I or II AVMs treated with stand-alone curative-intent intervention (resection, stereotactic radiosurgery [SRS] or endovascular embolisation). Bivariate and multivariable analyses were performed to identify patient and AVM characteristics predictive of complete obliteration or favourable outcome (complete obliteration without new permanent deficit or post-treatment haemorrhage).

**Results:**

A total of 522 patients were included (292 microsurgery, 152 SRS, 78 embolisation). Complete obliteration rates differed between microsurgery (95.9%), SRS (75.0%) and embolisation (71.8%) (*P* < .001); among patients classified as cured, digital subtraction angiography (DSA) confirmed obliteration in 96.1%, 75.9% and 52.4%, respectively. Favourable outcome was achieved in 89.4%, 64.5% and 67.9% (*P* < .001). In adjusted models for obliteration, elderly age (odds ratio [OR] 0.49, *P* = .022), larger nidus (OR 0.64, *P* = .005) and compacted morphology (OR 1.93, *P* = .019) were independent predictors overall; paediatric age predicted incomplete obliteration after microsurgery (OR 0.05, *P* = .045), and elderly age (OR 0.19, *P* = .001) and larger nidus (OR 0.59, *P* = .015) after SRS. For favourable outcome, SM grade II overall (adjusted OR [aOR] 0.58, *P* = .031) and elderly age after SRS (aOR 0.31, *P* = .019) were independent negative predictors.

**Discussion:**

Low-grade AVMs showed high rates of complete obliteration, though DSA-confirmed rates were substantially lower after embolisation, underscoring the importance of angiographic confirmation.

**Conclusion:**

The predictors identified in this study may help guide management when 2 or 3 of the primary treatment options carry clinical equipoise.

## Graphical Abstract

**Graphical Abstract:**
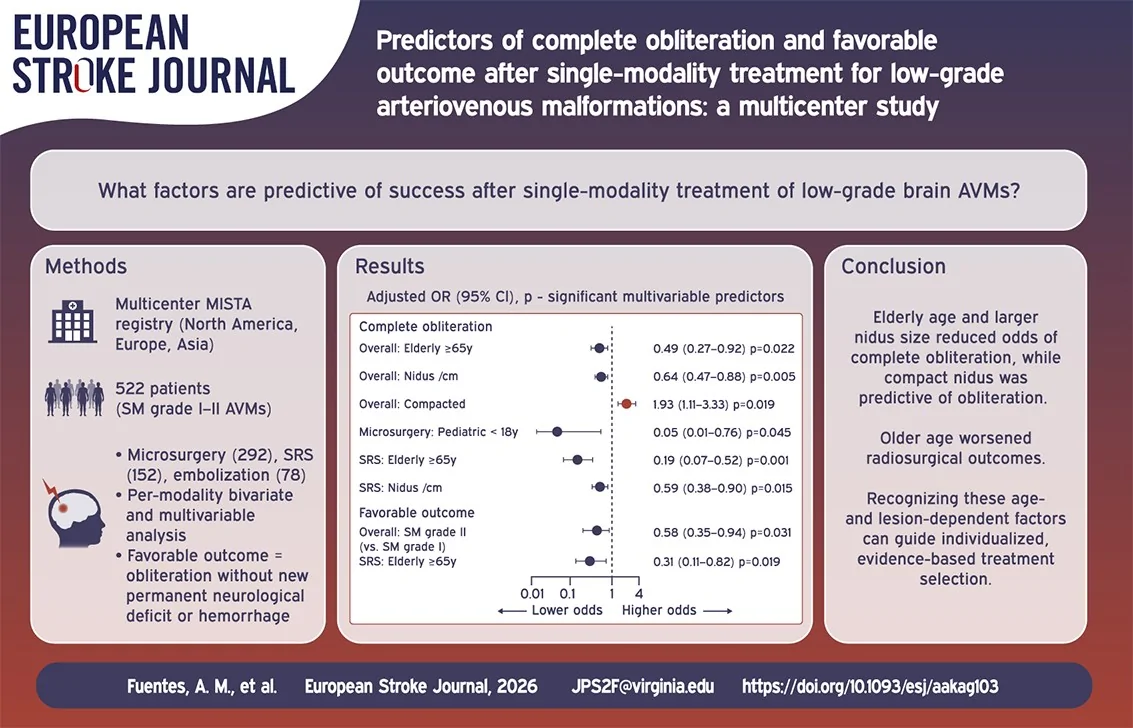

## Introduction

Brain arteriovenous malformations (AVMs) are complex vascular anomalies which shunt blood directly from the arterial to venous system through a tangle of vessels without an intervening capillary bed.^1^ Owing to elevated venous pressure, loss of normal vascular resistance and abnormal flow dynamics, AVMs are prone to rupture and their consequent haemorrhage represents a significant source of mortality and morbidity for neurosurgical patients.^1^^,^^2^ Management options include observation or treatment with either stand-alone or combination microsurgical resection, endovascular embolisation or stereotactic radiosurgery (SRS). Low-grade Spetzler–Martin (SM) I and II AVMs, which exhibit lower periprocedural risk with respect to size, location and venous drainage, are often considered as candidates for treatment.^3^^,^^4^ Furthermore, smaller AVMs compared to larger AVMs do more frequently present with haemorrhage.^5^^,^^6^ However, there remains debate on the optimal treatment modality for these lesions, as even low-grade AVMs carry a risk of adverse periprocedural event, with prior literature citing rates ranging from 3% to 21%.^6^^,^^7^ Despite previous evaluation of outcomes after treatment of low-grade AVMs, much remains unknown about specific patient and AVM characteristics that portend success between the different treatment modalities. This study was designed to identify predictive factors for outcomes within each treatment modality separately, rather than to perform head-to-head comparative effectiveness analysis between modalities.

## Patients and methods

### Study design and patient selection

This study was a retrospective, multicentre, observational study. The MISTA registry retrospectively collects standardised data from academic centres in North America, Europe and Asia, managing patients with AVMs. We included consecutive patients with low-grade (Spetzler–Martin grade I or II) brain AVMs who were treated between January 2010 and December 2023. The study was conducted in accordance with the Declaration of Helsinki and approved by the Institutional Review Board of each participating centre. Informed consent was waived due to the retrospective nature of the study.

Inclusion criteria for this study were: (1) patients with unruptured or ruptured Spetzler–Martin grade I or II brain AVMs, (2) receipt of treatment with curative intent by a single stand-alone modality comprising either microsurgical resection, SRS or endovascular embolisation, (3) at least 6 months of follow-up following resection or embolisation and (4) at least 3 years of follow-up following SRS unless complete radiographic obliteration was documented sooner. These follow-up thresholds reflect the expected time to treatment effect, with longer latency required for obliteration following SRS. Exclusion criteria were (1) Spetzler–Martin grades III–V AVMs, (2) an associated vascular malformation requiring additional treatment (eg, nidal aneurysm, developmental venous anomaly or cavernous malformation), (3) staged or multimodality treatment or (4) missing data on the primary or secondary outcome variables. The study adheres to the STROBE guidelines for the reporting of observational studies.^8^

Treatment with curative intent was verified based on documentation in the procedural report and pre-treatment multidisciplinary notes. For embolisation cases, only patients in whom the procedure was documented as being performed with the explicit goal of complete AVM eradication by embolisation alone were included. Cases where embolisation was intended as staged therapy prior to resection or radiosurgery were excluded. Patients who underwent embolisation and were later referred for additional therapy were captured under the “Retreatment” variable but were still analysed based on their initial treatment modality (intent-to-treat).

### Data collection and variable definitions

Patient demographics, clinical information, AVM characteristics, treatment details and outcomes were extracted from the MISTA registry database using a standardised excel sheet. Demographics included age at treatment, sex, race/ethnicity and family history of vascular malformations. Age was further categorised as paediatric (<18 years) or adult (≥18 years) and as elderly (≥65 years) or non-elderly (<65 years) for subgroup analyses. Information regarding medical comorbidities and use of anticoagulant or antiplatelet medications at presentation was collected. Neurological status at presentation was determined using the mRS, which ranges from 0 (no symptoms) to 6 (death).

Arteriovenous malformation characteristics were defined based on institutional review of available pre-treatment neuroimaging studies, comprising CTA, MRA and digital subtraction angiography (DSA). Details of AVM characteristics can be found in the supplemental materials.

From the full MISTA registry, we specifically included only patients who underwent single-modality, curative-intent treatment; patients managed with observation, palliative or adjunctive intent or planned multimodal/staged therapy were not part of this analytic cohort. Treatment selection, and the decision to treat rather than observe, was made by the treating physicians and institutional multidisciplinary neurovascular teams at each centre prior to enrolment, based on factors including AVM location and angioarchitecture, patient age and comorbidities, presenting symptoms and patient preferences. The clinical and angioarchitectural features most relevant to these treatment decisions (ruptured presentation, presenting mRS, seizure, deep location, venous stenosis and eloquent location) are reported by modality in Table 1.

**Table 1:** Baseline demographic and AVM characteristics by treatment modality for low-grade arteriovenous malformations.

Characteristic	Overall, *n* = 522 ^a^	Resection, *n* = 292 ^a^	Radiosurgery, *n* = 152 ^a^	Endovascular, *n* = 78 ^a^	*P*-value ^b^
***Age, years***	43.0 (26.0, 59.0)	45.0 (26.0, 60.3)	40.0 (23.0, 56.3)	44.5 (30.3, 62.8)	.14
***Paediatric classification***					.2
***Paediatric (<18 years)***	80 (15.3%)	45 (15.4%)	28 (18.4%)	7 (9.0%)	
***Adult (≥18 years)***	442 (84.7%)	247 (84.6%)	124 (81.6%)	71 (91.0%)	
***Elderly classification***					.2
***Non-elderly (<65 years)***	434 (83.1%)	242 (82.9%)	132 (86.8%)	60 (76.9%)	
***Elderly (≥65 years)***	88 (16.9%)	50 (17.1%)	20 (13.2%)	18 (23.1%)	
***Female***	260 (49.8%)	138 (47.3%)	82 (53.9%)	40 (51.3%)	.4
***Race***					
***Asian***	15 (3.1%)	12 (4.5%)	1 (0.7%)	2 (2.8%)	
***Black***	67 (14.0%)	38 (14.4%)	18 (12.7%)	11 (15.5%)	
***Hispanic***	80 (16.8%)	31 (11.7%)	33 (23.2%)	16 (22.5%)	
***White***	297 (62.3%)	171 (64.8%)	85 (59.9%)	41 (57.7%)	
***Other***	18 (3.8%)	12 (4.5%)	5 (3.5%)	1 (1.4%)	
***Unknown***	45	28	10	7	
***Family history***	14 (3.0%)	7 (2.7%)	7 (5.0%)	0 (0.0%)	.15
***Unknown***	51	32	11	8	
***Cardiovascular disease***	61 (12.7%)	31 (11.6%)	18 (12.7%)	12 (16.9%)	.5
***Unknown***	41	24	10	7	
***Hypertension***	135 (27.9%)	73 (27.0%)	37 (25.9%)	25 (35.2%)	.3
***Unknown***	38	22	9	7	
***Diabetes mellitus***	53 (11.0%)	32 (11.9%)	12 (8.5%)	9 (12.7%)	.5
***Unknown***	41	24	10	7	
***Anticoagulation***	23 (5.6%)	9 (4.1%)	6 (4.7%)	8 (13.6%)	.028
***Unknown***	114	71	24	19	
***Presentation mRS***					<.001
***0***	161 (31.6%)	53 (18.7%)	73 (49.3%)	35 (44.9%)	
***1***	142 (27.9%)	89 (31.4%)	44 (29.7%)	9 (11.5%)	
***2***	75 (14.7%)	51 (18.0%)	14 (9.5%)	10 (12.8%)	
***3***	62 (12.2%)	34 (12.0%)	13 (8.8%)	15 (19.2%)	
***4***	30 (5.9%)	24 (8.5%)	1 (0.7%)	5 (6.4%)	
***5***	39 (7.7%)	32 (11.3%)	3 (2.0%)	4 (5.1%)	
***Unknown***	13	9	4	0	
***Presentation mRS 0–2***	378 (72.4%)	193 (66.1%)	131 (86.2%)	54 (69.2%)	<.001
***Unknown***	13 (2.5%)	9 (3.1%)	4 (2.6%)	0 (0.0%)	
***Ruptured***	259 (49.6%)	171 (58.6%)	50 (32.9%)	38 (48.7%)	<.001
***Time of rupture***					<.001
***<24 h***	106 (46.9%)	75 (52.1%)	10 (22.7%)	21 (55.3%)	
***24 h–7d***	27 (11.9%)	20 (13.9%)	1 (2.3%)	6 (15.8%)	
***7d–14d***	16 (7.1%)	13 (9.0%)	1 (2.3%)	2 (5.3%)	
***>14d***	77 (34.1%)	36 (25.0%)	32 (72.7%)	9 (23.7%)	
***Unknown***	296	148	108	40	
***Preoperative seizure***	107 (21.4%)	53 (18.3%)	38 (28.6%)	16 (20.5%)	.057
***Unknown***	22	3	19	0	
***Location***					.2
***Infratentorial***	95 (18.2%)	46 (15.8%)	34 (22.4%)	15 (19.2%)	
***Supratentorial***	427 (81.8%)	246 (84.2%)	118 (77.6%)	63 (80.8%)	
***Eloquent location***	217 (41.6%)	109 (37.3%)	75 (49.3%)	33 (42.3%)	.051
***Deep location***	92 (17.8%)	41 (14.1%)	29 (19.3%)	22 (28.2%)	.013
***Unknown***	4	2	2	0	
***Nidus size (cm)***	1.7 (1.0, 2.3)	1.7 (1.0, 2.3)	1.7 (1.0, 2.4)	1.6 (1.2, 2.0)	.4
***Unknown***	14	8	3	3	
***Volume (cc)***	2.2 (0.9, 4.2)	2.0 (0.8, 3.7)	2.8 (1.3, 6.8)	2.1 (1.0, 3.1)	.006
***Unknown***	172	102	45	25	
***SM grade***					.016
***I***	211 (40.4%)	134 (45.9%)	51 (33.6%)	26 (33.3%)	
***II***	311 (59.6%)	158 (54.1%)	101 (66.4%)	52 (66.7%)	
***SM grade II subtype***					.4
***S1V0E1***	217 (69.8%)	109 (69.0%)	75 (74.3%)	33 (63.5%)	
***S1V1E0***	73 (23.5%)	36 (22.8%)	20 (19.8%)	17 (32.7%)	
***S2V0E0***	21 (6.8%)	13 (8.2%)	6 (5.9%)	2 (3.8%)	
***Compact***	356 (72.5%)	209 (76.0%)	102 (72.9%)	45 (59.2%)	.015
***Unknown***	31	17	12	2	
***Number of feeders***					.2
***Multiple***	261 (54.8%)	144 (52.6%)	80 (61.1%)	37 (52.1%)	
***Single***	215 (45.2%)	130 (47.4%)	51 (38.9%)	34 (47.9%)	
***Unknown***	46	18	21	7	
***Feeders’ diameter > 1 mm***	231 (57.8%)	156 (63.9%)	45 (46.9%)	30 (50.0%)	.007
***Unknown***	122	48	56	18	
***Number of draining veins***					.7
***Multiple***	132 (30.6%)	75 (29.8%)	37 (29.8%)	20 (35.7%)	
***Single***	300 (69.4%)	177 (70.2%)	87 (70.2%)	36 (64.3%)	
***Unknown***	90	40	28	22	
***Venous stenosis***	42 (9.8%)	26 (11.0%)	13 (10.2%)	3 (4.7%)	.3
***Unknown***	94	56	24	14	
***SRS modality***					NA
***GKRS***	97 (67.4%)	0 (NA%)	97 (67.4%)	0 (NA%)	
***LINAC***	31 (21.5%)	0 (NA%)	31 (21.5%)	0 (NA%)	
***Cyber-knife***	16 (11.1%)	0 (NA%)	16 (11.1%)	0 (NA%)	
***Unknown***	8	NA	8	NA	
***Prescription dose (Gy)***	20.0 (18.0, 21.0)	NA (NA, NA)	20.0 (18.0, 21.0)	NA (NA, NA)	NA
***Endovascular access***					NA
***Femoral***	55 (80.9%)	0 (NA%)	0 (NA%)	55 (80.9%)	
***Radial***	10 (14.7%)	0 (NA%)	0 (NA%)	10 (14.7%)	
***Other***	3 (4.4%)	0 (NA%)	0 (NA%)	3 (4.4%)	
***Unknown***	10	NA	NA	10	
***Embolisation material***					0.5
***NBCA***	9 (12.7%)	0 (NA%)	0 (NA%)	9 (12.7%)	
***Onyx***	62 (87.3%)	0 (NA%)	0 (NA%)	62 (87.3%)	
***Unknown***	7	NA	NA	7	
***Type of last image, n (%)***					>.9
***DSA***	276 (52.9%)	155 (53.1%)	79 (52.0%)	42 (53.8%)	
***MRA/CTA***	246 (47.1%)	137 (46.9%)	73 (48.0%)	36 (46.2%)	

Abbreviations: AVM = arteriovenous malformation; DSA = digital subtraction angiography; GKRS = gamma knife radiosurgery; LINAC = linear accelerator; NA = not applicable; NBCA = N-butyl cyanoacrylate; SM: Spetzler–Martin (grade); S1V0E1/S1V1E0/S2V0E0: Spetzler–Martin grade II Subtypes (size, venous drainage, eloquence); SRS = stereotactic radiosurgery.

^a^Median (25%, 75%); *n* (%)

^b^Kruskal–Wallis rank-sum test; Pearson’s chi-squared test; Fisher’s exact test.

### Outcome assessment

All patients were followed up per institutional protocols, which included clinical and imaging follow-up visits. Clinical follow-up typically consisted of a neurological examination and mRS assessment 1–3 months after treatment, and annually. Imaging follow-up was performed using MRA, CTA and/or DSA across all treatment modalities, depending on availability, institutional preference and clinical context. Follow-up imaging was typically obtained at 6- to 12-month intervals following treatment, whether resection, radiosurgery or endovascular embolisation, with DSA performed when available to confirm obliteration, particularly after resection or in cases with equivocal findings on non-invasive imaging.

The primary outcome was complete obliteration of the AVM at last follow-up imaging. Complete obliteration was assessed based on imaging modality. For DSA, obliteration was defined as the absence of residual AVM nidus and the absence of arteriovenous shunting, including early venous drainage. For MRA, obliteration was defined as the absence of flow voids, post-contrast enhancement or time-of-flight magnetic resonance angiogram signal. Partial obliteration was further categorised as near complete (90%–99%): tiny residual nidus or single remaining draining vein; substantial (50%–89%): significant reduction in nidus size but with persistent arteriovenous shunting or minimal (<50%): less than half of the original nidus obliterated. All imaging studies were interpreted by neuroradiologists at the respective institutions, and cases with indeterminate obliteration status were adjudicated by a second reader.

Secondary outcomes included mRS functional status at last follow-up. Good functional status was defined as mRS 0–2, and poor functional status was defined as mRS 3–5. Changes in mRS from presentation to last follow-up were categorised as improved (one or more point decrease in mRS score), stable (unchanged mRS score) or worse (increase in mRS score). Treatment-related complications were categorised as any complication (any adverse events attributed to the treatment), symptomatic complications (adverse events causing new neurological symptoms), permanent complications (neurological deficits present at last follow-up) and haemorrhagic complications (intracranial haemorrhage after treatment). Favourable outcome was defined as complete AVM obliteration and absence of post-treatment haemorrhage and/or permanent complications.

### Statistical analysis

All available cases meeting inclusion criteria were included in the analysis. Statistical analyses were performed using R Studio version 4.2.0 (R Foundation for Statistical Computing, Vienna, Austria) with packages including tidyverse, ggplot2, tableone, mice and rms.^9^ Categorical variables were reported as frequencies and percentages and compared using Pearson’s chi-squared test or Fisher’s exact test if the expected cell count was < 5. Continuous variables were tested for normality using the Shapiro–Wilk test and histograms, and variables that were not normally distributed were reported as medians with IQRs and compared using the Kruskal–Wallis rank-sum test, followed by Bonferroni correction for pairwise comparisons when significant.

To identify predictors of complete obliteration and favourable outcomes, bivariate and multivariable logistic regression models were performed for each treatment modality separately. Variables with *P* < .05 in the bivariate models were included in the respective multivariable models, and results were reported as ORs with 95% CIs. Obliteration outcomes were analysed using shift analysis (ordinal logistic regression) across 4 categories: complete, near complete (90%–99%), partial (50%–89%) and low (<50%). Treatment modality was the independent variable, and pairwise comparisons were performed using different reference groups. To reduce confounding by indication (bias in treatment selection), multivariable-adjusted analyses were performed using logistic regression models that included all baseline variables with *P* < .05 in bivariate comparisons between treatment groups. Results are reported as adjusted odds ratios (aORs) with 95% CIs. A *P*-value < .05 was considered statistically significant. Because DSA remains the reference standard for confirming AVM obliteration and non-invasive imaging (MRA/CTA) may differ in sensitivity across modalities (particularly after embolisation and SRS) we designated DSA-confirmed complete obliteration as a pre-specified co-primary outcome. Digital subtraction angiography-confirmed obliteration is reported descriptively alongside the all-imaging rate for each modality; modality-specific multivariable predictor models were not repeated within the DSA-confirmed subset, as further stratification by treatment modality reduced sample sizes (as low as 42 patients for embolisation) to a point where multivariable estimates would be unstable and unreliable.

Missing data were handled using complete-case analysis (listwise deletion), whereby patients with missing values for any variable included in a given regression model were excluded from that specific analysis only. Because anticoagulation status was included as a covariate in the favourable-outcome multivariable model and had a higher proportion of missing values than other covariates (114/522, 21.8%), models including this variable were additionally re-estimated using multiple imputation by chained equations (20 imputations) as a sensitivity analysis; the direction and significance of the associations were unchanged. To assess potential bias from missing data, we compared baseline characteristics between patients with complete vs incomplete data using chi-squared tests for categorical variables and Wilcoxon rank-sum tests for continuous variables. No significant differences were observed between these groups, suggesting that data were missing completely at random and that our complete-case approach did not introduce systematic bias.

## Results

### Baseline patient and AVM characteristics

The study sample included 522 patients with low-grade AVMs who underwent resection (*n* = 292, 56.0%), SRS (*n* = 152, 29.1%) and embolisation (*n* = 78, 14.9%) (Table 1). Paediatric patients (less than 18 years old) comprised 15.4%, 18.4% and 9.0% (*P* = .2); whereas elderly patients (at least 65 years old) comprised 17.1%, 13.2% and 23.1% (*P* = .2) of each of the microsurgery, SRS and embolisation groups, respectively. Differences in AVM characteristics included deep location, observed in 28.2% of embolisation cases vs 14.1% of microsurgical and 19.3% of SRS cases (*P* = .013); number of SM grade II lesions, observed in 66.4% of SRS cases vs 66.7% of embolisation and 54.1% of microsurgical cases (*P* = .016) and median AVM volume, which was 2.8 cc for SRS vs 2.0 cc for microsurgical and 2.1 cc for embolisation cases (*P* = .006). Follow-up imaging modality to assess obliteration varied across treatment groups: DSA was used in 53.1% of microsurgical cases, 52.0% of radiosurgical cases and 53.8% of embolisation cases, while the remainder were followed with MRA or CTA (Table 1). Median imaging follow-up differed by modality (microsurgery 12.0 months, SRS 39.0 months, embolisation 13.0 months; *P* < .001), reflecting the longer latency to obliteration after SRS (Table 2).

**Table 2:** Summary of clinical, radiographic and safety outcomes across treatment modalities for patients with low-grade arteriovenous malformations.

Characteristic	Overall, *n* = 522 ^a^	Resection, *n* = 292 ^a^	Radiosurgery, *n* = 152 ^a^	Endovascular, *n* = 78 ^a^	*P*-value ^b^
***Obliteration***					**<.001**
***Last follow-up complete obliteration confirmed by DSA*^c^**	231/276 (83.7%)	149/155 (96.1%)	60/79 (75.9%)	22/42 (52.4%)	
***Last follow-up complete obliteration*^d^**	450 (86.2%)	280 (95.9%)	114 (75.0%)	56 (71.8%)	
***Last follow-up (90%–99%) obliteration*^d^**	22 (4.2%)	6 (2.1%)	5 (3.3%)	11 (14.1%)	
***Last follow-up (50%–89%) obliteration*^d^**	*31 (5.9%)*	*4 (1.4%)*	*20 (13.2%)*	*7 (9.0%)*	
***Last follow-up (<50%) obliteration*^d^**	*19 (3.6%)*	*2 (0.7%)*	*13 (8.6%)*	*4 (5.1%)*	
***Functional outcomes***					
***mRS score (0–2) at last follow-up***	416 (90.2%)	214 (87.3%)	145 (97.3%)	57 (83.6%)	**<.001**
** *Unknown***	61	47	3	11	
***mRS score change at last follow-up (*vs *presentation)***					**<.001**
** *Same***	203 (44.2%)	89 (36.6%)	82 (55.8%)	32 (46.4%)	
** *Better***	208 (45.3%)	131 (53.9%)	52 (35.4%)	25 (36.2%)	
** *Worse***	48 (10.5%)	23 (9.5%)	13 (8.8%)	12 (17.4%)	
** *Unknown***	63	49	5	9	
***Complications***					
***All complications***	108 (20.7%)	57 (19.5%)	37 (24.3%)	14 (17.9%)	.4
***Symptomatic complications***	74 (14.2%)	42 (14.4%)	24 (15.8%)	8 (10.3%)	.5
***Permanent complications***	37 (7.1%)	19 (6.5%)	15 (9.9%)	3 (3.8%)	.2
***Haemorrhagic complication***	15 (3.2%)	6 (2.1%)	7 (5.6%)	2 (2.9%)	.2
***Unknown***	48	12	26	10	
***Favourable outcomes*** ^**e**^	412 (78.9%)	261 (89.4%)	98 (64.5%)	53 (67.9%)	**<.001**
***Retreatment***	31 (5.9%)	15 (5.1%)	11 (7.2%)	5 (6.4%)	.6
***Retreatment type*** ^**f**^					**.013**
***Resection***	13/31 (41.9%)	9/15 (60.0%)	2/11 (18.2%)	2/5 (40.0%)	
***Stereotactic radiosurgery***	14/31 (45.2%)	4/15 (26.7%)	9/11 (81.8%)	1/5 (20.0%)	
***Endovascular embolisation***	4/31 (12.9%)	2/15 (13.3%)	0/11 (0.0%)	2/5 (40.0%)	
***Mortality***	12 (2.2%)	7 (2.4%)	2 (1.3%)	3 (3.8%)	.5
***Mortality related to procedure*** ^**g**^	2 (0.4%)	2 (0.7%)	0 (0.0%)	0 (0.0%)	>.9
***Length of hospital stay (days)***	4.0 (1.0, 9.0)	6.0 (4.0, 12.0)	0.0 (0.0, 1.0)	6.0 (2.0, 11.0)	**<.001**
***Length of imaging follow-up (months)***	23.0 (6.0, 46.8)	12.0 (6.0, 34.0)	39.0 (37.4, 60.0)	13.0 (7.2, 26.9)	**<.001**
***Length of clinical follow-up (months)***	24.0 (7.0, 45.0)	12.5 (6.0, 25.0)	39.0 (37.5, 61.5)	22.5 (6.0, 47.0)	**<.001**

Abbreviations: AVM = arteriovenous malformation; DSA = digital subtraction angiography.

^a^ *n* (%); median (25%,75%).

^b^Pearson’s chi-squared test; Fisher’s exact test; Kruskal–Wallis rank-sum test.

^c^Subset of patients with follow-up imaging by DSA.

^d^Obliteration assessed using any imaging modality: DSA, MRA or CTA.

^e^Defined as complete AVM obliteration and absence of post-treatment haemorrhage and/or permanent complications.

^f^Patients were categorised based on initial treatment modality (intent-to-treat). Retreatment reflects subsequent interventions due to incomplete obliteration or recurrence and does not alter initial group assignment.

^g^Attributable to haemorrhagic complications.

bold face denotes a p < 0.05 which was deemed statistically significant.

### Obliteration rates

**Figure 1:**
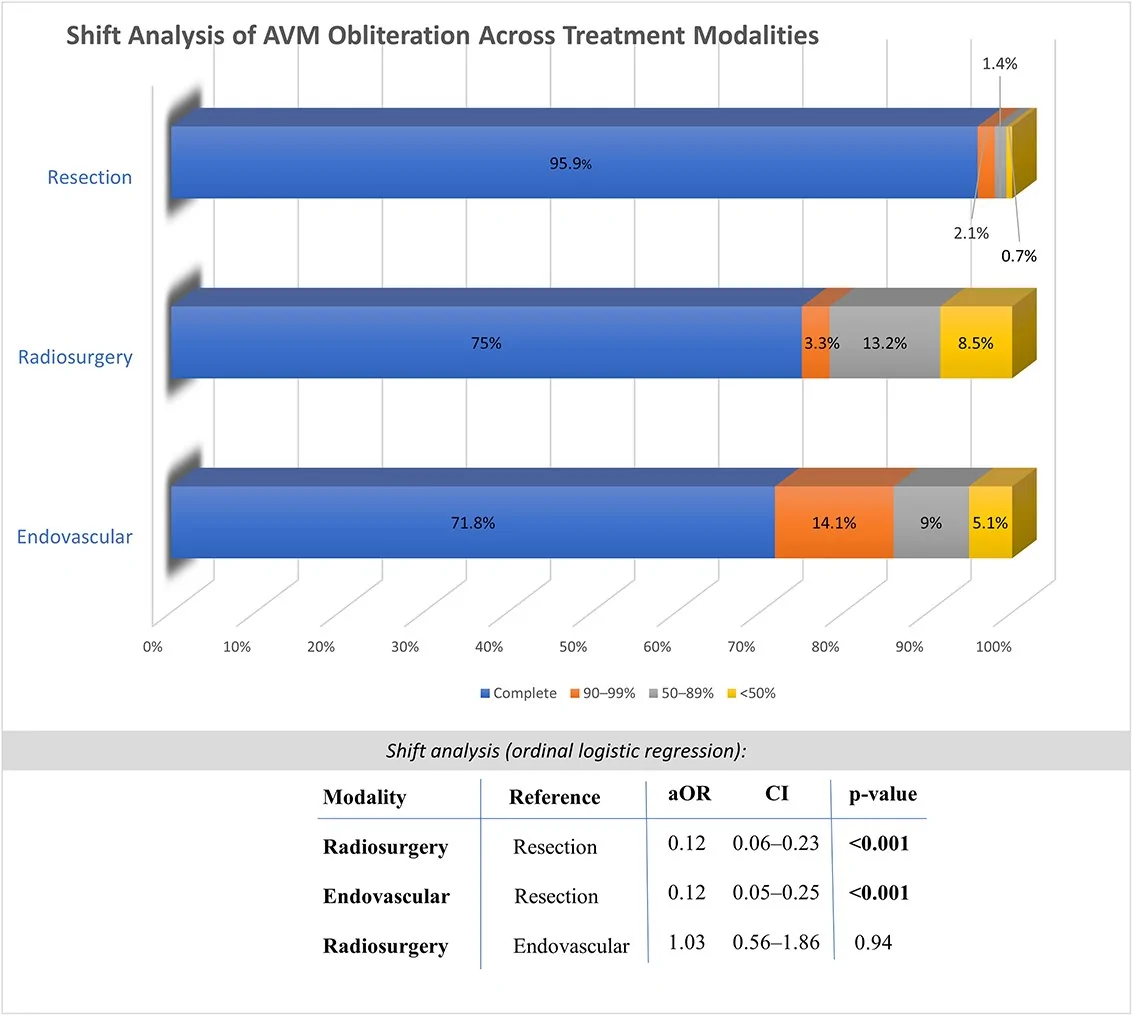
AVM obliteration outcomes by treatment modality. Stacked bars show the distribution of obliteration levels after resection, radiosurgery and endovascular therapy. Resection had the highest complete obliteration rate (95.9%), followed by radiosurgery (75%) and endovascular (71.8%). Shift analysis below shows both radiosurgery and endovascular were significantly less effective than resection (*P* < .001), with no difference between radiosurgery and endovascular (*P* = .94). Abbreviation: AVM = arteriovenous malformation.

**Figure 2:**
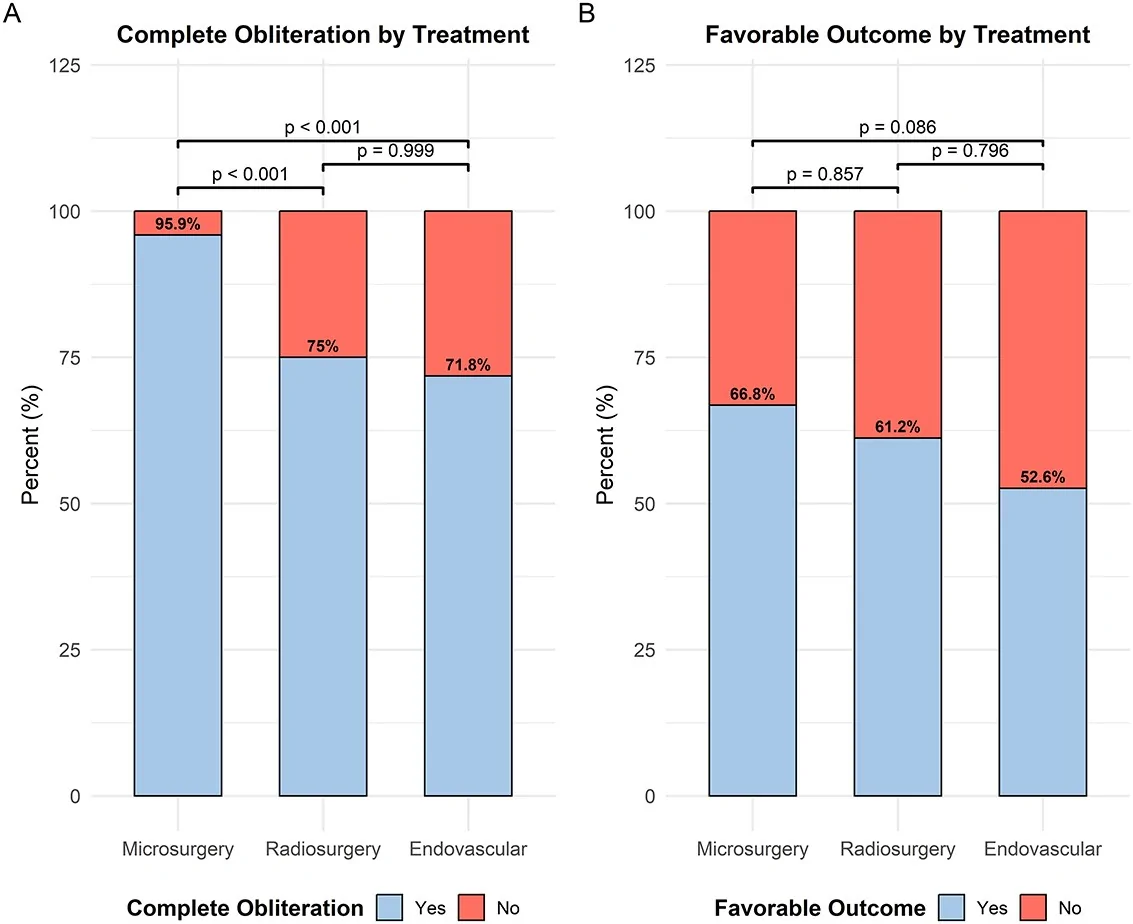
AVM outcomes by treatment modality*.* (A) Microsurgery achieved the highest rate of complete obliteration (95.9%) compared to radiosurgery (75%) and endovascular therapy (71.8%) (*P* < .001), (B) Favourable outcomes were also highest with microsurgery (89.4%), followed by endovascular therapy (67.9%) and radiosurgery (64.5%). Abbreviation: AVM = arteriovenous malformation.

Complete obliteration rates were 95.9% after microsurgery, 75.0% after radiosurgery and 71.8% after embolisation (*P* < .001). On shift analysis using ordinal logistic regression, both radiosurgery and embolisation were associated with significantly lower odds of achieving a greater degree of obliteration compared to microsurgical resection (aOR 0.12 for both; *P* < .001) (Figures 1 and 2). No significant difference was observed between radiosurgery and embolisation (aOR 1.03, *P* = .94).

In bivariate analysis, several patient, AVM and treatment factors were significantly associated with obliteration across all 3 treatment modalities (Table S1). Detailed description of these findings can be found in the supplemental materials.

**Figure 3:**
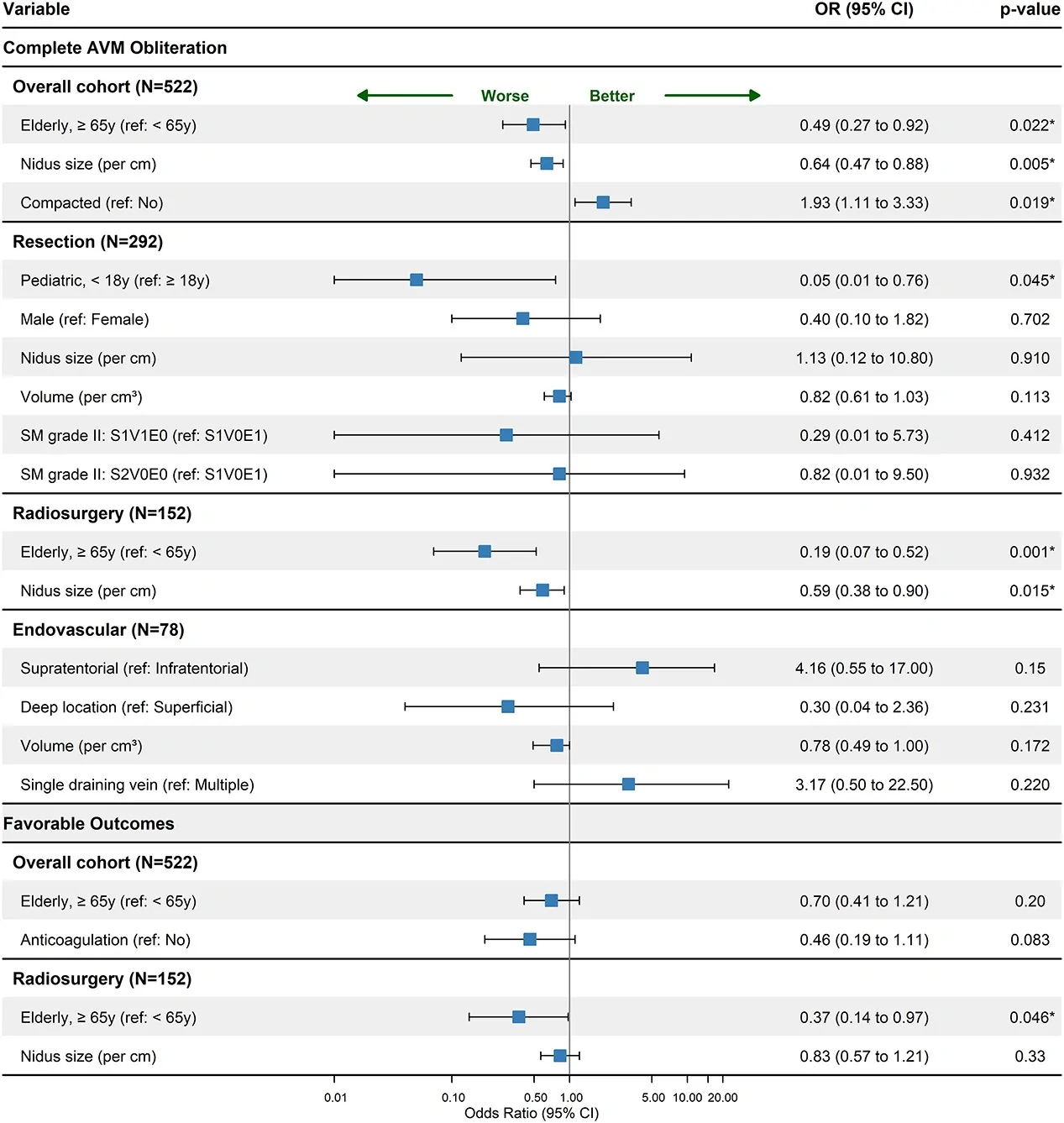
Multivariable (adjusted) predictors of complete AVM obliteration and favourable outcomes across all treatment modalities and subgroup analyses*.* Forest plots display aORs with 95% CIs for factors associated with complete obliteration (top) and favourable outcomes (bottom). In the overall cohort, older age (≥65), larger nidus size and compacted AVMs were significantly associated with lower odds of complete obliteration. Subgroup analysis showed similar trends, with elderly patients and larger nidus size predicting poorer obliteration following radiosurgery. Older age also predicted reduced odds of favourable outcomes in the radiosurgery group (*P* < .05). Abbreviations: aOR = adjusted odds ratio; AVM = arteriovenous malformation.

In multivariable analysis, for the overall cohort, elderly age (OR 0.49; 95% CI, 0.27–0.92; *P* = .022), larger nidus size (OR 0.64; 95% CI, 0.47–0.88; *P* = .005) and compacted morphology (OR 1.93; 95% CI, 1.11–3.33; *P* = .019) remained significantly associated with obliteration. Elderly age and larger nidus size were negative predictors (OR < 1), whereas compacted morphology was a positive predictor (OR > 1). After microsurgery, paediatric age (OR 0.05, *P* = .045) remained a significant negative predictor of complete obliteration. After SRS, elderly age (OR 0.19, *P* = .001) and larger nidus size (OR 0.59, *P* = .015) were independent negative predictors of complete obliteration (Figure 3). No predictors remained significant after embolisation in multivariable analyses.

Digital subtraction angiography-confirmed complete obliteration was achieved in 96.1% of microsurgery cases (149/155), 75.9% of SRS cases (60/79) and 52.4% of embolisation cases (22/42) among patients classified as cured on any imaging modality (Table 2).

### Functional outcomes and favourable outcomes

Compared to baseline presentation, mRS scores at last follow-up improved in 53.9% (*n* = 131), 35.4% (*n* = 52) and 36.2% (*n* = 25) of cases after microsurgery, SRS and embolisation, respectively; and mRS scores worsened in 9.5% (*n* = 23), 8.8% (*n* = 13) and 17.4% (*n* = 12), after microsurgery, SRS and embolisation, respectively (*P* < .001) (Table 2). The rate of favourable outcome differed significantly across treatment groups, occurring in 78.9% of the overall cohort (*n* = 412) and in 89.4% (*n* = 261) after microsurgery, 64.5% (*n* = 98) after SRS and 67.9% (*n* = 53) after embolisation (*P* < .001) (Table 2; Figure 2; Figure S1).

In the overall cohort, Spetzler–Martin grade II (vs grade I) was a significant negative predictor of favourable outcome on bivariate analysis (OR 0.51; 95% CI, 0.32–0.79; *P* = .004). No other variables were significantly associated with favourable outcome in the overall cohort. In treatment-specific analyses, elderly age remained a significant negative predictor of favourable outcome after SRS (OR 0.31; 95% CI, 0.11–0.81; *P* = .018) (Table S2; Figure 3). After embolisation, deep location was a significant negative predictor of favourable outcome (OR 0.25; 95% CI, 0.09–0.71; *P* = .010), whereas supratentorial location (OR 4.41; 95% CI, 1.38–15.1; *P* = .014) and a single draining vein (OR 3.39; 95% CI, 1.03–11.8; *P* = .047) were positive predictors. No significant predictor of favourable outcome was identified after microsurgery. In multivariable analysis, Spetzler–Martin grade II remained an independent negative predictor of favourable outcome in the overall cohort (aOR 0.58; 95% CI, 0.35–0.94; *P* = .031), and elderly age remained independent after SRS (aOR 0.31; 95% CI, 0.11–0.82; *P* = .019). After embolisation, no factor retained significance following adjustment, and no independent predictor was identified after microsurgery.

### Retreatment for residual or recurrent AVM

Retreatment for residual or recurrent AVM occurred in 31 patients (5.9%), with comparable rates across groups: 5.1% (15/292) after microsurgery, 7.2% (11/152) after SRS and 6.4% (5/78) after embolisation (*P* = .6). The modality used for retreatment differed by initial treatment, predominantly repeat resection after microsurgery (9/15), repeat SRS after SRS (9/11) and a mixed approach after embolisation (Table 2).

## Discussion

Though the primary aim of our study is to identify predictors of complete obliteration and favourable outcome after treatment of low-grade AVM, we also present outcome differences between the different treatment modalities. Concordant with our findings, several prior studies have demonstrated superior obliteration rates after microsurgery compared to SRS for low-grade AVMs.^10–12^ Additionally, obliteration rates after upfront embolisation are lower than for microsurgery or SRS.^13^ In our cohort, complete obliteration after stand-alone embolisation was achieved in 71.8% of cases overall and in 52.4% of the subset with DSA-confirmed follow-up. These rates should be interpreted within the context of a highly selected population in whom embolisation was deliberately chosen as stand-alone curative-intent therapy. They are not directly comparable with pragmatic randomised or all-comer series such as the TOBAS trial, in which the endovascular arm was prospectively randomised, obliteration was confirmed exclusively by DSA and lesions triaged to planned multimodal therapy were excluded at the outset, design differences that systematically bias any cross-study comparison towards the more highly selected cohort.^14^ Rather than indicating superior efficacy, these results most likely reflect this selection, together with the use of modern embolic agents and detailed angioarchitectural characterisation at the time of case selection. Our registry captured granular angioarchitectural features, including number of arterial feeders, number of draining veins and presence of venous stenosis. In bivariate analysis, AVMs with single draining veins, in supratentorial location, and smaller volume were more likely to be completely obliterated following embolisation. Deep location was a significant negative predictor of obliteration after embolisation (OR 0.18; 95% CI, 0.06–0.53; *P* = .002) and predicted lower odds of favourable outcome (OR 0.25; 95% CI, 0.09–0.71; *P* = .010). This probably can be explained that deep-seated AVMs are more often supplied by small perforating or choroidal vessels that are difficult to catheterise and embolise safely, and the higher proportion of deep-seated lesions in the embolisation group (28.2% vs 14.1% microsurgery and 19.3% SRS) may partly explain its lower obliteration rate relative to resection. Additionally, the majority of embolised AVMs were compact (59.2%), and most were treated with Onyx (87.3%), a non-adhesive liquid embolic agent that allows for deeper, more controlled nidus penetration. Our findings are consistent with recent literature. A systematic review of embolisation outcomes in low-grade AVMs reported obliteration rates of approximately 60% in selected patients, and a recent multicentre cohort study reported an obliteration rate of 57.8% achieved primarily with modern Onyx-based embolisation techniques, with confirmation of obliteration based on DSA, MRA or CTA, similar to our methodology.^13^^,^^15^ Like our cohort, the majority of cases in that study were treated with Onyx, reflecting current endovascular practice. The slightly lower obliteration rate compared to our study (57.8% vs 71.8%) may be partially explained by differences in AVM grade distribution; their cohort included Spetzler–Martin grades I–III, whereas our analysis focused exclusively on grades I and II, which are inherently more amenable to complete obliteration. These findings, alongside our own, underscore the evolving curative potential of embolisation when applied to appropriately selected low-grade AVMs using modern embolic agents and advanced techniques.

In the microsurgery group, paediatric age was the only significant predictor of incomplete obliteration in adjusted analyses. While this finding appears to contrast with prior literature demonstrating comparable obliteration rates between paediatric and adult patients across all AVM grades, it likely reflects differences in study design and outcome definitions rather than a contradiction in surgical efficacy.^16^ Notably, our study defined obliteration according to long-term follow-up angiography, whereas previous studies often relied on early postoperative imaging, which may not capture delayed AVM recurrence, a phenomenon well documented in paediatric populations. The paediatric brain is known to have higher angiogenic potential, and recurrence after angiographically confirmed resection has been reported in up to 10.4% of paediatric cases.^17–19^ As such, the lower obliteration rates observed in our paediatric cohort may be more accurately attributed to true recurrence over time, rather than incomplete initial resection. Moreover, it is plausible that diffuse nidus morphology, which complicates complete resection and is associated with higher residual rates, may be more prevalent in paediatric AVMs, although this was not formally assessed in our cohort and warrants further investigation. In some cases, surgeons may also adopt a more conservative resection strategy in young patients, particularly when AVMs are located in eloquent or deep brain regions, prioritising preservation of neurological function over aggressive removal. This clinical nuance could further contribute to residual AVM tissue despite technically successful surgery.

Both nidus size and patient age feature in scoring systems predicting outcomes after SRS for AVMs.^20^^,^^21^ In our SRS group, elderly age and larger nidus size were significant negative predictors of AVM obliteration on adjusted analyses, concordant with our findings from the entire cohort. Smaller AVM volume and presence of single draining vein have inconsistently been shown to predict complete AVM obliteration after SRS.^1^^,^^6^^,^^22^^,^^23^ The relationship between age and AVM obliteration is variable in studies of all AVM grades, with some but not all studies finding a significant association between older age and lower obliteration rates.^24^^,^^25^ It has been hypothesised that the reduced response to SRS in elderly patients may be associated with age-related differences in the radiobiological profile of the AVM nidus, and this warrants exploration in future studies.^25^^,^^26^

Elderly age was the only predictor of unfavourable outcome identified after SRS in adjusted analysis. While this contradicts prior literature suggesting comparable outcomes for elderly and non-elderly patients after SRS, the lower obliteration rates post-SRS in the elderly patient cohort of the present study are a potential contributing factor to these observed worse outcomes.^25^^,^^26^ Though it trended towards significance, elderly age was not a significant predictor of favourable outcome among our entire cohort, suggesting this factor is more influential specifically after SRS. Elderly age was not a predictor of unfavourable outcome in the microsurgical cohort. A significant relationship between advanced age and worse functional outcomes after AVM resection has been established in several prior studies.^27–30^ However, unlike these prior analyses, the current study utilised elderly vs non-elderly age classification, which may owe to the difference in significant findings.

In this highly selected cohort in whom embolisation was chosen as stand-alone curative-intent therapy, complete obliteration was achieved in 71.8% overall and in 52.4% of those with DSA confirmation. Although no predictor of obliteration survived multivariable analysis in the embolisation arm, likely reflecting its limited size (*n* = 78), deep location predicted both lower obliteration and lower odds of favourable outcome, whereas supratentorial location and a single draining vein were associated with more favourable outcomes. These findings support embolisation as a reasonable minimally invasive option in appropriately selected patients when microsurgery or SRS are not feasible or cannot be offered but an indication for treatment persists. Larger cohorts are needed to confirm these angioarchitectural associations.

Among the entire cohort, factors related to AVM morphology including nidus size and compactness were predictive of complete obliteration but not of favourable outcome, demonstrating that radiographic cure does not necessarily equate to clinical benefit. In addition, these morphological features were not consistently demonstrated to be predictors of complete obliteration in the treatment-specific analyses. These AVM characteristics likely vary in the degree of impact they have in the nuances of treatment involved in these different modalities, which involve significant differences in how they are planned and technically implemented.

Treatment-related complications did not differ significantly across modalities. Any complication occurred in 19.5% of microsurgery, 24.3% of SRS and 17.9% of embolisation patients (*P* = .4); symptomatic complications in 14.4%, 15.8% and 10.3% (*P* = .5) and permanent neurological deficits in 6.5%, 9.9% and 3.8% (*P* = .2), respectively. Post-treatment haemorrhage was uncommon in all groups (microsurgery 2.1%, SRS 5.6%, embolisation 2.9%; *P* = .2). Although not statistically significant, the numerically higher rate of permanent deficit after SRS and the lower rate after embolisation are consistent with the distinct risk profiles of each modality: microsurgery and SRS carry procedural and radiation-related risks to eloquent tissue, whereas the principal risk of stand-alone embolisation in this selected cohort was incomplete obliteration rather than new deficit. Because none of these comparisons was statistically significant and the groups differed at baseline, the numerical differences should not be interpreted as evidence of modality-specific safety differences.

## Limitations

Despite rigorous review of procedural documentation, misclassification of treatment intent is possible, particularly in embolisation cases that achieved cure despite originally being intended as adjunctive. However, we mitigated this by excluding any patients who had documented staged or planned multimodal therapy. Additionally, our use of an intent-to-treat framework, whereby patients who required retreatment were still analysed within their initial modality group, reduces the risk of overestimating embolisation efficacy. Treatment-specific analyses avoid directly attributing between-group outcome differences to treatment modality, but they remain vulnerable to selection bias, residual confounding, and treatment-by-indication effects within each selected cohort. In the SRS cohort, additional follow-up may have resulted in a higher percentage of patients achieving obliteration as the latency period after SRS is typically 1–5 years. In addition, it is possible that small AVM residuals were not seen on follow-up MRI or CTA, impacting accuracy of reported obliteration in patients without follow-up angiography.

Our results are contingent upon the accuracy and completeness of reported data, which was largely based upon medical chart abstraction. Imaging was interpreted by neuroradiologists at each participating centre without central adjudication, and the registry did not capture formal dual-read or inter-rater agreement metrics; we therefore cannot quantify inter-observer reliability or exclude differential misclassification across modalities. The discordance between all-imaging and DSA-confirmed obliteration (particularly after embolisation, where 52.4% of cases classified as cured were DSA-confirmed) illustrates this concern and is the reason DSA-confirmed obliteration was analysed as a co-primary outcome. This heterogeneity may similarly affect secondary endpoints extracted by chart abstraction, and these limitations should temper any use of these data for cross-modality treatment selection. For the same reason, we did not repeat multivariable obliteration models within modality-specific DSA-confirmed subsets, as the resulting sample sizes were too small to support stable estimation; DSA-confirmed obliteration is therefore reported descriptively rather than modelled. Because treatment was at the discretion of the treating physician and institutional multidisciplinary team, and no standardised decision model or flowchart was applied, treatment selection was influenced not only by lesion characteristics but also by local expertise, equipment and institutional traditions; centres may have varied in treatment protocols and follow-up, introducing treatment-selection bias. Consequently, the predictor findings and any comparative signals may not generalise to centres with differing endovascular expertise or SRS platforms, and should be validated in other settings. Compact morphology predicted obliteration in the overall cohort but in no individual modality. Because compact AVMs were concentrated in the microsurgery arm (76.0% vs 59.2% embolisation, *P* = .015), the highest-obliteration modality, this pooled association likely reflects confounding by indication rather than a true modality-independent effect. The paediatric-age association with incomplete obliteration after microsurgery was based on few events (45/292) and may reflect delayed angiographic recurrence rather than incomplete resection; it should therefore be interpreted cautiously as hypothesis-generating rather than a definitive treatment predictor. Furthermore, the power of our analysis was limited by the relatively small number of patients in each treatment arm, particularly the endovascular cohort (*n* = 78); the absence of significant multivariable predictors of obliteration after embolisation should therefore be interpreted as reflecting limited statistical power rather than a definitive lack of association. We excluded patients treated with multimodal or staged approaches (such as preoperative embolisation and subsequent resection), which limits the generalisability of our findings to only those patients who received stand-alone therapy.

## Conclusions

In this multicentre study of low-grade brain AVMs, we identified patient and AVM characteristics associated with complete obliteration and favourable outcomes across the 3 major treatment modalities: microsurgery, stereotactic radiosurgery (SRS) and endovascular embolisation. These age-related associations may help inform counselling but require confirmation, particularly the paediatric microsurgery finding. On multivariable analysis, larger nidus size and older age likewise predicted lower obliteration, whereas compact morphology predicted higher obliteration, and Spetzler–Martin grade II independently predicted less favourable outcome, factors that can help calibrate expectations at the time of treatment selection. While microsurgery achieved the highest obliteration rates for technically resectable SM I–II AVMs, SRS and embolisation remain valuable alternatives in selected anatomical or clinical scenarios. Ongoing investigation is warranted to refine individualised, evidence-based treatment strategies for patients with low-grade AVMs.

## Supplementary Material

Supplemental_material_aakag103
