# Supplementary material for: Predictors of complete obliteration and favourable outcome after single-modality treatment for low-grade arteriovenous malformations: a multicentre study
Source: Eur Stroke J. 2026 Aug 31;11(8):aakag103. doi: 10.1093/esj/aakag103 (PMC13528858; doi:10.1093/esj/aakag103)
Supplement: Supplementary file 1 [file supplemental_material_aakag103.docx]

**Predictors of complete obliteration and favorable outcome after single-modality treatment for low-grade arteriovenous malformations: A multicenter study**

**Supplemental Materials.**

***Details of AVM characteristic definitions of MISTA registry:***

The Spetzler-Martin grading system is used to assign AVMs a risk of surgical morbidity based on nidus size (<3 cm, 3-6 cm, >6 cm), eloquence of adjacent brain, and pattern of venous drainage (superficial vs. deep).^3^ Grade II AVMs were further subclassified based on component factors: S1V0E1 (small size, superficial venous drainage, eloquent location), S1V1E0 (small size, deep venous drainage, non-eloquent location), or S2V0E0 (medium size, superficial venous drainage, non-eloquent location). Eloquent brain regions were defined according to Spetzler-Martin criteria as sensorimotor, language, or visual cortex; hypothalamus; thalamus; internal capsule; brainstem; cerebellar peduncles; and deep cerebellar nuclei. Nidus location was categorized as supratentorial or infratentorial, and lobar or deep. Deep location was defined as basal ganglia, thalamus, or brainstem. AVM nidus size was measured as maximum diameter in centimeters, as directly reported from DSA or MRI. Nidus volume was not always directly reported. Therefore, nidus volume was calculated using the formula (A × B × C)/2 where A, B, and C were the maximum diameters in each of the three dimensions. Nidus morphology was defined as compact (clear borders with no intervening normal brain) or diffuse (poorly defined borders with intervening normal brain). Angioarchitectural features included number of feeding arteries (single or multiple), feeding artery diameter ($\leq$1 mm or >1 mm), presence of perforator arterial feeders, number of draining veins (single or multiple), presence of venous stenosis or ectasia, and presence of intranidal or flow-related aneurysms.

***Detailed findings of bivariate analysis assessing factors associated with complete obliteration***

For overall cohort, elderly age (OR 0.51, 95% CI 0.29–0.92, p=0.022) and larger nidus size (OR 0.67, 95% CI 0.50–0.89, p=0.005) were significant negative predictors of complete obliteration, while compacted nidus morphology (OR 1.92, 95% CI 1.12–3.26, p=0.016) was a positive predictor. Among the 358 AVMs treated with microsurgery, significant negative predictors of complete obliteration were pediatric age (OR 0.16, 95% CI 0.05-0.54, p=0.003), male gender (OR 0.21, 95% CI 0.03-0.82, p=0.048), larger nidus size (OR 0.50, 95% CI 0.27-0.95, p=0.030), larger AVM volume (OR 0.90, 95% CI 0.81-1.01, p=0.046), and S2V0E0 subtype (OR 0.10, 95% CI 0.01-0.92, p=0.030). Among the 151 AVMs treated with SRS, significant negative predictors of complete obliteration included elderly age (OR 0.21, 95% CI 0.08-0.56, p=0.002), larger nidus size (OR 0.63, 95% CI 0.41-0.94, p=0.025), and superficial location (OR 0.28, 95% CI 0.06-0.87, p=0.050). Among the 77 AVMs treated with embolization, significant negative predictors of complete obliteration included infratentorial location (OR 0.17, 95% CI 0.05-0.56, p=0.004), multiple draining veins (OR 0.24, 95% CI 0.07-0.83, p=0.026), larger AVM volume (OR 0.75, 95% CI 0.54-0.93, p=0.037), and deep location (OR 0.18, 95% CI 0.06-0.53, p=0.002).

**Supplemental Tables and Figures.**

**Table S1.** Bivariate analysis of factors associated with complete AVM obliteration across all treatment modalities and in subgroup analyses.

**Table S2:** Bivariate analysis of factors associated with Favorable Outcomes across all treatment modalities and in subgroup analyses.

**Figure S1.** *Bar graph showing the proportion of favorable vs. unfavorable outcomes across treatment modalities.*

| **Table S1.** Bivariate analysis of factors associated with complete AVM obliteration across all treatment modalities and in subgroup analyses. | | | | | | | | | | | | | | | | | | | | | |
| --- | --- | --- | --- | --- | --- | --- | --- | --- | --- | --- | --- | --- | --- | --- | --- | --- | --- | --- | --- | --- | --- |
|  | | | **Overall** | | | | | **Resection** | | | | **Radiosurgery** | | | **Endovascular** | | | | | | |
| **Characteristic** | | | **OR***^1^* | **95% CI***^1^* | | **p-value** | | **OR***^1^* | **95% CI***^1^* | | **p-value** | **OR***^1^* | **95% CI***^1^* | **p-value** | **OR***^1^* | | | **95% CI***^1^* | | | **p-value** |
| ***Age, years*** | | | 1.00 | 0.99, 1.01 | | 0.80 | | 1.03 | 1.00, 1.06 | | 0.085 | 0.99 | 0.97, 1.01 | 0.49 | 1.00 | | | 0.97, 1.02 | | | 0.83 |
| ***Elderly, ≥ 65y (vs < 65y)*** | | | 0.51 | 0.29, 0.92 | | **0.022** | | 1.03 | 0.26, 6.87 | | 0.97 | 0.21 | 0.08, 0.56 | **0.002** | 0.73 | | | 0.24, 2.38 | | | 0.58 |
| ***Pediatric, < 18y (vs ≥ 18y)*** | | | 0.64 | 0.35, 1.24 | | 0.16 | | 0.16 | 0.05, 0.54 | | **0.003** | 1.00 | 0.40, 2.74 | >0.99 | 0.98 | | | 0.19, 7.23 | | | 0.98 |
| ***Male [vs Female]*** | | | 0.89 | 0.54, 1.46 | | 0.64 | | 0.21 | 0.03, 0.82 | | **0.048** | 0.81 | 0.39, 1.69 | 0.57 | 1.55 | | | 0.58, 4.32 | | | 0.39 |
| ***Cardiovascular disease*** | | | 0.58 | 0.30, 1.18 | | 0.11 | | 1.18 | 0.21, 22.2 | | 0.87 | 0.52 | 0.19, 1.53 | 0.22 | 0.57 | | | 0.16, 2.14 | | | 0.38 |
| ***Hypertension*** | | | 0.96 | 0.56, 1.72 | | 0.89 | | 0.86 | 0.23, 4.07 | | 0.83 | 0.68 | 0.30, 1.57 | 0.35 | 2.34 | | | 0.78, 8.07 | | | 0.15 |
| ***Diabetes Meletus*** | | | 1.72 | 0.72, 5.09 | | 0.27 | | 1.23 | 0.22, 23.0 | | 0.85 | 1.91 | 0.48, 12.9 | 0.42 | 1.67 | | | 0.36, 11.9 | | | 0.55 |
| ***Anticoagulation*** | | | 0.60 | 0.23, 1.88 | | 0.33 | | — | — | |  | 0.18 | 0.02, 0.96 | 0.052 | 2.92 | | | 0.46, 57.0 | | | 0.34 |
| ***Seizure*** | | | 1.24 | 0.64, 2.60 | | 0.54 | | 2.54 | 0.48, 47.0 | | 0.38 | 1.11 | 0.44, 3.07 | 0.84 | 1.91 | | | 0.54, 9.05 | | | 0.35 |
| ***Ruptured*** | | | 1.55 | 0.94, 2.59 | | 0.089 | | 0.27 | 0.04, 1.05 | | 0.10 | 1.28 | 0.58, 2.93 | 0.55 | 2.02 | | | 0.74, 5.78 | | | 0.17 |
| ***Supratentorial location*** | | | 1.47 | 0.79, 2.63 | | 0.20 | | 1.07 | 0.16, 4.25 | | 0.93 | 0.58 | 0.20, 1.44 | 0.27 | 5.77 | | | 1.77, 20.2 | | | **0.004** |
| ***Eloquent location*** | | | 0.87 | 0.53, 1.45 | | 0.59 | | 3.09 | 0.80, 20.4 | | 0.15 | 0.73 | 0.35, 1.52 | 0.40 | 1.08 | | | 0.40, 3.02 | | | 0.88 |
| ***Deep location*** | | | 0.65 | 0.37, 1.22 | | 0.16 | | 0.82 | 0.21, 5.44 | | 0.80 | 3.53 | 1.15, 15.5 | 0.050 | 0.18 | | | 0.06, 0.53 | | | **0.002** |
| ***Nidus size (cm)*** | | | 0.67 | 0.50, 0.89 | | **0.005** | | 0.50 | 0.27, 0.95 | | **0.030** | 0.63 | 0.41, 0.94 | **0.025** | 0.72 | | | 0.32, 1.57 | | | 0.40 |
| ***Volume (cm^3^)*** | | | 1.00 | 0.98, 1.01 | | 0.51 | | 0.90 | 0.81, 1.01 | | **0.046** | 1.00 | 0.99, 1.03 | 0.90 | 0.75 | | | 0.54, 0.93 | | | **0.037** |
| ***SM grade II*** | | | 0.61 | 0.35, 1.03 | | 0.068 | | 0.84 | 0.24, 2.68 | | 0.76 | 0.75 | 0.33, 1.64 | 0.49 | 0.68 | | | 0.21, 1.93 | | | 0.48 |
| ***SM grade II subtype*** | | |  |  | |  | |  |  | |  |  |  |  |  | | |  | | |  |
| *S1V0E1* | | | — | — | |  | | — | — | |  | — | — |  | — | | | — | | |  |
| *S1V1E0* | | | 0.88 | 0.44, 1.87 | | 0.73 | | 0.21 | 0.03, 1.29 | | 0.090 | 2.20 | 0.65, 10.1 | 0.24 | 0.69 | | | 0.20, 2.49 | | | 0.56 |
| *S2V0E0* | | | 0.43 | 0.16, 1.29 | | 0.11 | | 0.10 | 0.01, 0.92 | | **0.030** | 0.39 | 0.07, 2.24 | 0.27 | 0.38 | | | 0.01, 10.2 | | | 0.50 |
| ***Compact*** | | | 1.92 | 1.12, 3.26 | | **0.016** | | 2.77 | 0.78, 9.52 | | 0.10 | 1.10 | 0.45, 2.53 | 0.83 | 2.21 | | | 0.81, 6.18 | | | 0.12 |
| ***Number of feeders*** | | |  |  | |  | |  |  | |  |  |  |  |  | | |  | | |  |
| *Multiple* | | | — | — | |  | | — | — | |  | — | — |  | — | | | — | | |  |
| *Single* | | | 0.89 | 0.53, 1.51 | | 0.66 | | 0.50 | 0.13, 1.70 | | 0.28 | 0.67 | 0.31, 1.45 | 0.30 | 1.43 | | | 0.48, 4.46 | | | 0.53 |
| ***Feeders’ diameter > 1mm*** | | | 0.90 | 0.47, 1.68 | | 0.75 | | 1.44 | 0.35, 5.58 | | 0.60 | 0.44 | 0.15, 1.21 | 0.12 | 0.61 | | | 0.19, 1.88 | | | 0.39 |
| ***Number of draining veins*** | | |  |  | |  | |  |  | |  |  |  |  |  | | |  | | |  |
| *Multiple* | | | — | — | |  | | — | — | |  | — | — |  | — | | | — | | |  |
| *Single* | | | 1.30 | 0.73, 2.27 | | 0.36 | | 0.58 | 0.09, 2.38 | | 0.50 | 0.99 | 0.42, 2.27 | 0.99 | 4.09 | | | 1.21, 14.9 | | | **0.026** |
| ***Venous stenosis*** | | | 2.44 | 0.85, 10.3 | | 0.15 | | 1.12 | 0.20, 21.1 | | 0.92 | 5.04 | 0.94, 93.6 | 0.13 | 0.84 | | | 0.08, 18.7 | | | 0.89 |
| ***Prescription dose (Gy)*** | | | — | — | |  | | — | — | |  | 1.00 | 0.84, 1.19 | >0.99 | — | | | — | | |  |
|  |  |  |  | |  | |  | | |  | | | |  |  |  |  | |  |  |  |

| **Table S2:** Bivariate analysis of factors associated with Favorable Outcomes across all treatment modalities and in subgroup analyses. | | | | | | | | | | | | | | | | | |
| --- | --- | --- | --- | --- | --- | --- | --- | --- | --- | --- | --- | --- | --- | --- | --- | --- | --- |
|  | | | | **Overall** | | | **Resection** | | | | | **Radiosurgery** | | | **Endovascular** | | |
| **Characteristic** | | | | **OR***^1^* | **95% CI***^1^* | **p-value** | **OR***^1^* | | **95% CI***^1^* | | **p-value** | **OR***^1^* | **95% CI***^1^* | **p-value** | **OR***^1^* | **95% CI***^1^* | **p-value** |
| ***Age, years*** | | | | 1.00 | 0.99, 1.01 | 0.74 | 1.01 | | 0.99, 1.02 | | 0.49 | 1.00 | 0.98, 1.01 | 0.69 | 1.00 | 0.98, 1.02 | 0.95 |
| ***Elderly, ≥ 65y (vs < 65y)*** | | | | 0.66 | 0.39, 1.13 | 0.12 | 1.08 | | 0.43, 3.34 | | 0.88 | 0.31 | 0.11, 0.81 | **0.018** | 0.67 | 0.23, 2.09 | 0.48 |
| ***Pediatric, < 18y (vs ≥ 18y)*** | | | | 0.91 | 0.52, 1.64 | 0.73 | 0.58 | | 0.25, 1.55 | | 0.25 | 1.47 | 0.62, 3.80 | 0.40 | 0.60 | 0.12, 3.25 | 0.52 |
| ***Male [vs Female]*** | | | | 0.88 | 0.58, 1.34 | 0.55 | 0.68 | | 0.31, 1.43 | | 0.32 | 0.70 | 0.36, 1.36 | 0.29 | 1.32 | 0.51, 3.49 | 0.57 |
| ***Cardiovascular disease*** | | | | 0.82 | 0.45, 1.59 | 0.55 | 1.71 | | 0.47, 11.0 | | 0.48 | 0.96 | 0.35, 2.77 | 0.94 | 0.40 | 0.11, 1.46 | 0.16 |
| ***Hypertension*** | | | | 0.85 | 0.53, 1.38 | 0.50 | 0.63 | | 0.28, 1.49 | | 0.28 | 0.73 | 0.34, 1.59 | 0.43 | 1.86 | 0.64, 5.91 | 0.27 |
| ***Diabetes Meletus*** | | | | 1.21 | 0.61, 2.63 | 0.61 | 1.09 | | 0.35, 4.80 | | 0.89 | 1.25 | 0.37, 4.88 | 0.73 | 0.95 | 0.23, 4.87 | 0.95 |
| ***Anticoagulation*** | | | | 0.49 | 0.21, 1.26 | 0.12 | 0.83 | | 0.14, 15.9 | | 0.87 | 0.12 | 0.01, 0.75 | 0.053 | 1.25 | 0.25, 9.19 | 0.80 |
| ***Seizure*** | | | | 1.24 | 0.72, 2.24 | 0.45 | 1.52 | | 0.56, 5.31 | | 0.46 | 2.09 | 0.89, 5.35 | 0.10 | 0.74 | 0.24, 2.43 | 0.60 |
| ***Ruptured*** | | | | 1.42 | 0.93, 2.18 | 0.10 | 0.64 | | 0.28, 1.39 | | 0.28 | 1.26 | 0.62, 2.63 | 0.53 | 1.68 | 0.65, 4.51 | 0.29 |
| ***Supratentorial location*** | | | | 1.44 | 0.85, 2.38 | 0.17 | 1.66 | | 0.63, 3.95 | | 0.27 | 0.58 | 0.24, 1.33 | 0.21 | 4.41 | 1.38, 15.1 | **0.014** |
| ***Eloquent location*** | | | | 0.68 | 0.44, 1.04 | 0.073 | 1.09 | | 0.51, 2.45 | | 0.82 | 0.60 | 0.31, 1.18 | 0.14 | 0.71 | 0.27, 1.86 | 0.49 |
| ***Deep location*** | | | | 0.71 | 0.42, 1.21 | 0.19 | 0.80 | | 0.31, 2.50 | | 0.68 | 2.00 | 0.82, 5.38 | 0.14 | 0.25 | 0.09, 0.71 | **0.010** |
| ***Nidus size (cm)*** | | | | 0.82 | 0.63, 1.05 | 0.11 | 0.75 | | 0.48, 1.19 | | 0.21 | 0.82 | 0.56, 1.18 | 0.28 | 0.76 | 0.35, 1.62 | 0.47 |
| ***Volume (cm^3^)*** | | | | 1.00 | 0.98, 1.01 | 0.51 | 0.93 | | 0.84, 1.03 | | 0.10 | 1.00 | 0.99, 1.02 | 0.85 | 0.80 | 0.60, 1.20 | 0.065 |
| ***SM grade II*** | | | | 0.51 | 0.32, 0.79 | **0.004** | 0.53 | | 0.23, 1.13 | | 0.11 | 0.66 | 0.32, 1.35 | 0.26 | 0.52 | 0.17, 1.47 | 0.23 |
| ***SM grade II subtype*** | | | |  |  |  |  | |  | |  |  |  |  |  |  |  |
| *S1V0E1* | | | | — | — |  | — | | — | |  | — | — |  | — | — |  |
| *S1V1E0* | | | | 1.01 | 0.55, 1.91 | 0.97 | 0.47 | | 0.17, 1.36 | | 0.15 | 2.11 | 0.73, 7.05 | 0.19 | 1.05 | 0.31, 3.70 | 0.94 |
| *S2V0E0* | | | | 0.66 | 0.26, 1.83 | 0.40 | 0.37 | | 0.10, 1.85 | | 0.18 | 0.70 | 0.12, 4.02 | 0.68 | 0.57 | 0.02, 15.3 | 0.70 |
| ***Compact*** | | | | 0.99 | 0.63, 1.54 | 0.95 | 0.81 | | 0.29, 1.97 | | 0.66 | 1.20 | 0.55, 2.56 | 0.65 | 1.99 | 0.75, 5.33 | 0.17 |
| ***Number of feeders*** | | | |  |  |  |  | |  | |  |  |  |  |  |  |  |
| *Multiple* | | | | — | — |  | — | | — | |  | — | — |  | — | — |  |
| *Single* | | | | 0.99 | 0.63, 1.54 | 0.95 | 0.65 | | 0.29, 1.42 | | 0.28 | 0.98 | 0.48, 2.03 | 0.96 | 1.18 | 0.42, 3.38 | 0.76 |
| ***Feeders’ diameter > 1mm*** | | | | 1.18 | 0.71, 1.94 | 0.53 | 1.34 | | 0.57, 3.05 | | 0.48 | 0.75 | 0.32, 1.75 | 0.51 | 0.73 | 0.23, 2.20 | 0.57 |
| ***Number of draining veins*** | | | |  |  |  |  | |  | |  |  |  |  |  |  |  |
| *Multiple* | | | | — | — |  | — | | — | |  | — | — |  | — | — |  |
| *Single* | | | | 1.31 | 0.80, 2.11 | 0.28 | 0.65 | | 0.23, 1.58 | | 0.37 | 1.55 | 0.71, 3.39 | 0.27 | 3.39 | 1.03, 11.8 | **0.047** |
| ***Venous stenosis*** | | | | 1.26 | 0.59, 3.00 | 0.58 | 0.99 | | 0.31, 4.39 | | 0.99 | 1.45 | 0.44, 5.59 | 0.56 | 0.90 | 0.08, 20.2 | 0.94 |
| ***Prescription dose (Gy)*** | | | | — | — |  | — | | — | |  | 0.95 | 0.81, 1.12 | 0.55 | — | — |  |
|  |  |  |  |  |  |  |  |  | |  |  |  |  |  |  |  |  |

**Figure S1.** *Bar graph showing the proportion of favorable vs. unfavorable outcomes across treatment modalities.*
